# A Unique Case of Plunging Dermoid Cyst in an Elderly Male: A Case Report

**DOI:** 10.7759/cureus.44874

**Published:** 2023-09-07

**Authors:** Trupti V Gaikwad, Sunil S Mishra, Anuj P Maini, Sukanya Das, Arunima Sarma

**Affiliations:** 1 Oral Medicine and Radiology, Dr. D.Y. Patil Dental College and Hospital, Dr. D. Y. Patil Vidyapeeth, Pune, IND; 2 Oral Medicine and Radiology, Government Dental College, Dibrugarh, IND

**Keywords:** developmental cysts, diagnostic challenges, submandibular region, swelling, submental, dermoid cyst, plunging ranula

## Abstract

Dermoid cysts are the least commonly occurring developmental cysts in the oral and maxillofacial region. They may be congenital or acquired and are seen as asymptomatic swellings that are slow and progressive. It is very difficult to differentiate plunging ranulas from plunging dermoid cysts as both of them have very similar clinical features. However, since both entities have different treatment strategies, it is important to differentiate one from the other. A 57-year-old male patient reported to the Department of Oral Medicine and Radiology with a large swelling in the submental region. To the best of our knowledge, the present case report is the first one showing such an extensive lesion of plunging dermoid cyst mimicking plunging ranula in an elderly male patient. The report mainly focuses on the diagnostic challenges faced to reach the final diagnosis.

## Introduction

Dermoid cysts are developmental pathologies that initiate in the various organs or tissues due to the enclosure of tissues from various bases (endoblastic, mesoblastic, or ectoblastic) formed by a fault in the union of the lateral embryonal mesenchymal tissues (usually the first and the second arch) through the fifth week of embryonal growth [1]. It is comparatively less common in the oral and maxillofacial region. However, 6.5% of dermoid cysts found in the oral and maxillofacial region are seen in the oral cavity. Most often they are found in the peri-orbital area [2]. They typically exist as a non-symptomatic swelling, with steady and progressing growth, not usually linking more than one anatomic location [3].

Plunging dermoid cysts and lateral dermoid cysts are comparatively rare entities that pose a diagnostic challenge as they usually resemble plunging ranulas in appearance [4]. When these swellings in the floor of the mouth dissect through the mylohyoid muscle and produce swelling within the neck, they are referred to as plunging ranulas or plunging dermoid cysts. Ranulas can be managed by the procedure of marsupialization and this procedure has a small level of recurrence, while dermoid cysts are usually managed by the procedure of surgical excision [3]. Since the treatment approach is different for both lesions, it is important to diagnose these lesions correctly. We present a case of a 57-year-old male patient who presented with a large swelling in the submental region appearing as a dermoid cyst. 

## Case presentation

A 57-year-old male presented to the Department of Oral Medicine and Radiology with the chief complaint of difficulty in swallowing and change in voice for two months. The patient also had difficulty in protruding his tongue since then. He had no history of hypertension, diabetes, or cardiological, renal, hepatic, pulmonary, or thyroid problems, and was not on any medications. The family history and past dental history were also non-significant. All teeth in all four quadrants were present. They showed no findings of dental caries, fractures, or abrasion. Generalized attrition was present in almost all the teeth along with mild stains and calculus. The patient had no adverse habits of tobacco, cigarette, areca nut, or alcohol consumption. 

**Figure 1:**
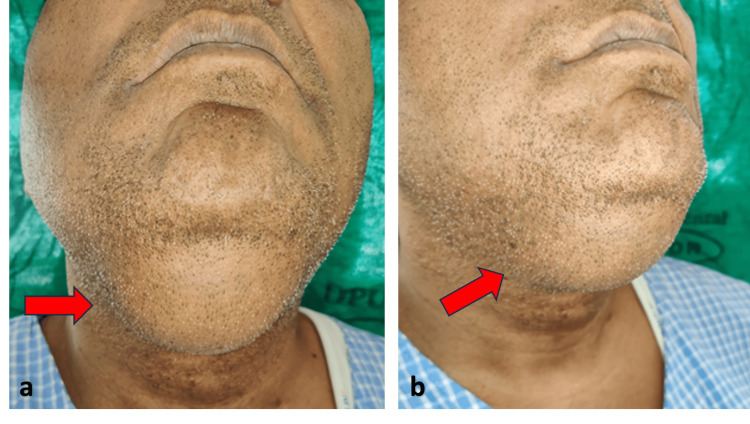
Round to oval shaped swelling in the submental region with no obvious color change and measuring approximately 5x4x3 cm in size.

On extra-oral examination, a solitary diffuse swelling was seen below the chin in the midline (Figure 1). It extended along the inferior border of the mandible on the left side to the right side. It was round to oval in shape. The size was approximately 5x6 cm. No obvious color change was noted over the swelling. The surrounding skin showed no abnormality. On palpation, there was no local rise in the temperature. The swelling was painless and soft in consistency. It was non-fluctuant, mobile, and compressible. There was a history of swelling, which was present from his childhood and was much smaller in size. The patient had not taken any treatment during his childhood for the same. It gradually increased in size to up to the present.

**Figure 2:**
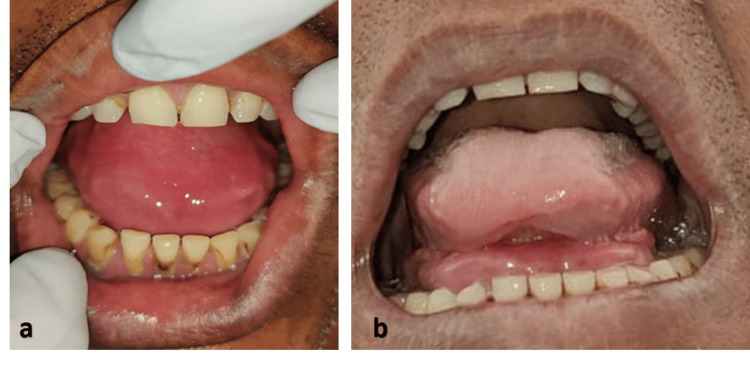
(a) Evident erythematous swelling with no tongue seen as it touched the palate; (b) Tongue protruding outwards and swelling seen below the tongue

On intraoral examination, a solitary swelling was seen on the midline region covering the entire floor of the mouth (Figure 2). It had no effect on the flow of saliva. It extended from the left side of the ridge to the right side. It was round to oval in shape measuring approximately 5x4x3 cm in size. The swelling appeared to be slightly erythematous. It was soft in consistency and painless. It was fluctuant, compressible, and mobile. No pus discharge was noted on compression of the swelling. On bimanual palpation, mobility of the swelling was present.

A provisional diagnosis of plunging ranula was given and lymphatic malformation, dermoid cyst, epidermoid cyst, thyroglossal duct cyst, hemangioma, benign salivary gland tumor, and cystic hygroma were considered as differential diagnosis. The patient was advised to undergo ultrasonography (USG) of the submental region to know if the lesion was vascular. In the USG, a well-defined thick-walled (2.1 mm) anechoic cystic lesion of size measuring approximately 38x46x46 mm with thick internal moving echoes and minimal peripheral vascularity was noted in the sublingual space, which was suggestive of a simple infected ranula.

As further anatomic detail and connection of the lesion to surrounding structures were lacking, a computed tomography (CT) scan was advised. The CT scan showed a well-defined large hypodense lesion seen in the midline on the floor of the mouth/ root of the tongue, extending inferiorly in the submental region with a contoured bulge measuring 87x48x43 mm. The anterior portion of the lesion showed multiple fat-density areas (sack of marble appearance). The swelling seemed to displace genioglossus laterally and mylohyoid muscles inferno-laterally. The coronal and sagittal views showed a dumbbell-shaped lesion with one bulge projecting into the oral cavity and the other one below the inferior border of the mandible (Figure 2a).

**Figure 3:**
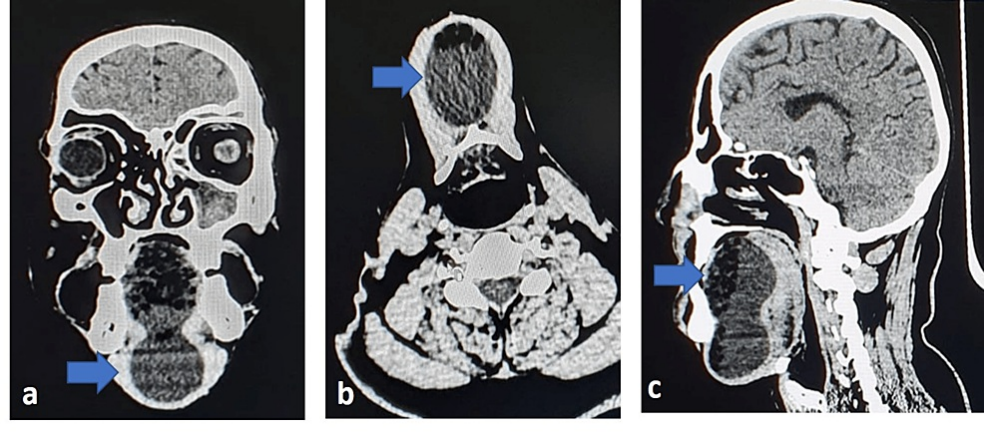
Computed tomography images of the lesion a) Coronal view showing dumbbell shaped lesion with multiple fat densities b) Axial view showing the inferior bulge with multiple fat densities c) Sagittal view showing dumbbell shaped hypodensity with multiple fat densities in the anterior portion

The axial view showed the inferior bulge as a round-shaped hypodensity with multiple fat densities in the anterior portion (Figure 2b). The superior bulge showed multiple round fat densities in the coronal view and similar fat densities were also seen in the anterior portion of the superior bulge and some portion of the inferior bulge anteriorly in the sagittal view (Figure 2c). All the findings were suggestive of a dermoid cyst. Epidermoid cyst, thyroglossal duct cyst, ranula, and lymphatic malformation were considered radiographic differential diagnoses.

Fine needle aspiration cytology (FNAC) was done, which showed abundant cyst macrophages along with scattered neutrophils and lymphocytes. The background was hemorrhagic. Occasional nucleate and anucleate squames were also seen. No malignant cells were noted. All these findings were suggestive of a benign cystic lesion.

**Figure 4:**
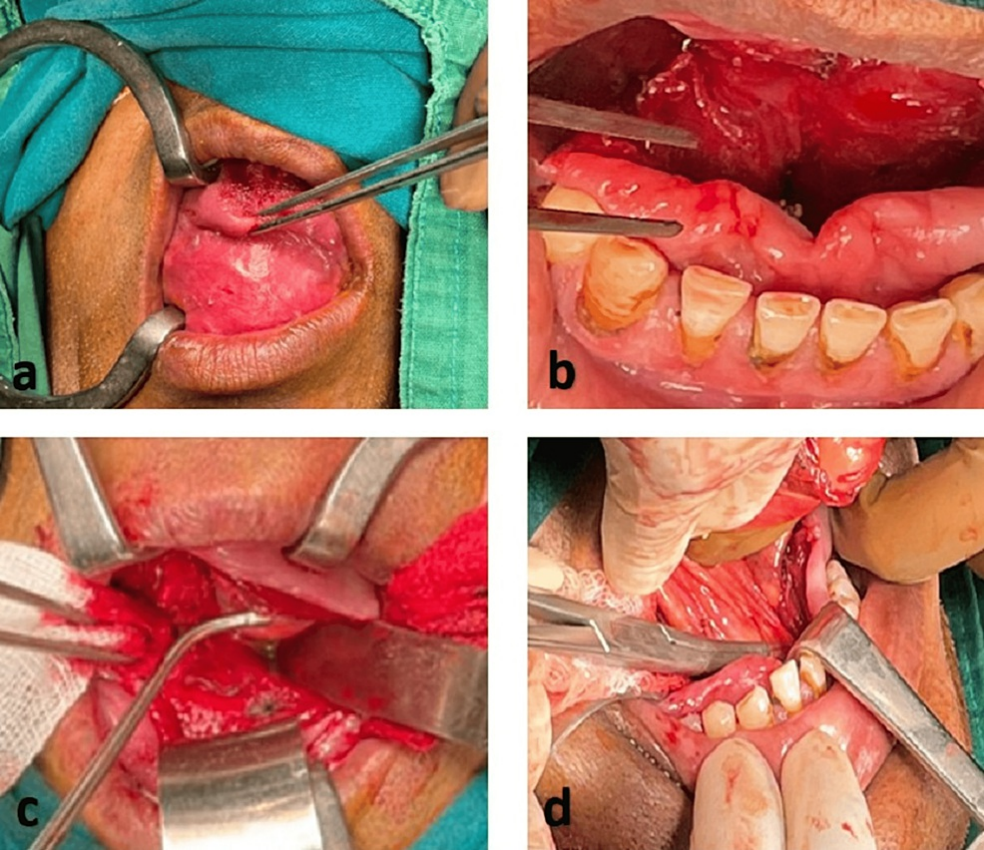
Intra-operative images of the patient: (a) Retraction of the tongue; (b) Incision surrounding the lesion; (c) Excision of the lesion; (d) Separation of the lesion

The patient underwent complete blood count, liver function, and blood sugar level (fasting and post-prandial) tests before undergoing the surgery. All the values were found to be within the normal limits. Complete excision of the cyst was done under general anesthesia. A mid-line incision was taken along the longitudinal ventral surface of the tongue to the floor of the mouth and by careful dissection, the cyst was separated out (Figure 4). Postoperatively, the patient was given tablet Pan-40 mg, once a day for two weeks, Tablet Ultracet, twice a day for two weeks, and tablet Chymoral Forte, three times a day for three weeks.

**Figure 5:**
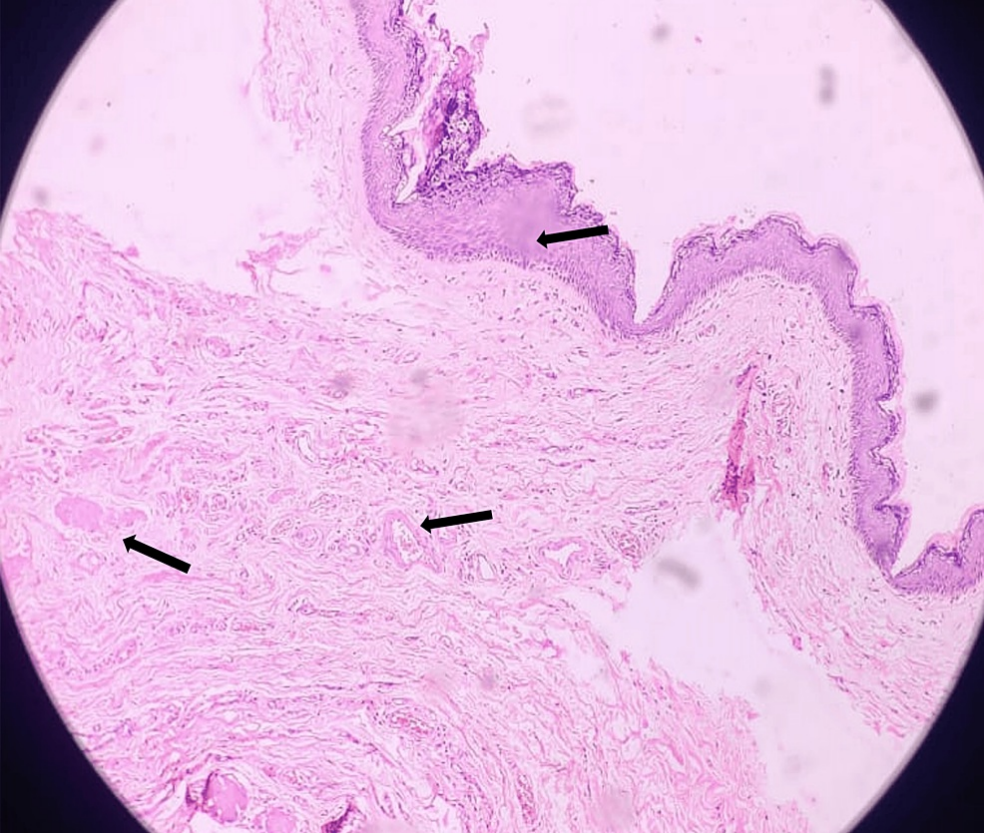
Hematoxylin and eosin-stained section (10x) showing cystic lining composed of squamous epithelium with keratin debris and sebaceous glands with associated hair follicles. There appears to be no evidence of malignancy. There is evidence of chronic inflammation and giant cell reaction.

A single, greyish-white, soft tissue piece measuring approximately 6x3x3 cm showing clay-like pultaceous material was sent for histopathological examination. Under the hematoxylin and eosin stain (10x magnification) multiple sections of the encapsulated lesion were studied and showed soft tissue lined by keratinized stratified squamous epithelium. Underlying connective tissue stroma showed numerous thick-walled blood vessels, chronic inflammatory cells, and muscle cells. Some sections also showed multiple multi-nucleated foreign bodies like giant cells along with adipocytes and sebaceous glands. No atypical or malignant features were seen (Figure 5). All the findings were indicative of a dermoid cyst. 

**Figure 6:**
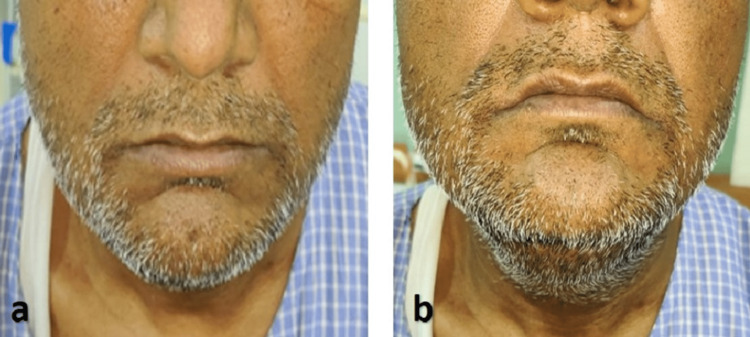
Postoperative images showing (a) frontal view with (b) postoperative edema

The patient was followed up after one week. Reduction in the size of the swelling was noted along with some post-operative edema (Figure 6).

## Discussion

Dermoid cysts are typically made up of lipid material with tissue peripherally organized and fluid arranged centrally. They are benign and they rarely tend to rupture [5]. Meyer restructured the notion of dermoid cysts to define various histopathological alternatives: true dermoid cyst, teratoid cyst, and epidermoid cyst [6].

True dermoid cysts are hollows that are lined with epithelium that show keratinization and with a recognizable covering on the cystic barrier. The lining of the epidermoid cysts is with simple squamous epithelium with a fibrous barrier and no structures devoted. The teratoid cyst’s lining differs from a simple squamous one to a ciliate respiratory epithelium that contains various derivatives of endoderm, mesoderm, or ectoderm. All three histopathological variants have a thick, cheesy, keratinous, and greasy-looking material [1].

The etiology of dermoid cysts can be typically congenital, or acquired, or it may arise from the rest of the totipotent cells that are exiled from the blastomere [7,8]. It initiates congenitally because of the trapped mid-line embryonal cells of the first and second branchial arches or it might evolve from the tuberculum impar. Acquired cysts arise because of the distressing epithelial cell implantation occurring due to accident, surgery, or as a result of the obstruction of a duct of the sebaceous gland. There is no gender predilection. Also, these types of cysts are seen commonly in individuals in the age group of 15-35 years, in a certain phase of maximum epithelial action [9].

The differential diagnoses of sublingual swellings include ranulas, thyroglossal duct cysts, cystic hygroma, lymphatic malformations, dermoid cysts, epidermoid cysts, heterotopic gastrointestinal cysts, and duplication fore-gut cysts [10]. Ranula and thyroglossal duct cysts were excluded as a final diagnosis in the current case as these cysts do not exhibit areas of fat attenuation on CT scans. This feature helped us to differentiate them from the dermoid cyst. Cystic hygromas and lymphatic malformations show fluid density on USG and CT scans. Therefore, these two were also excluded from the differential diagnosis. On histopathological examination, hair follicles and sebaceous glands were present, which helped exclude epidermoid cysts also from the differential diagnosis list. Hence, a final diagnosis of dermoid cyst was made.

A similar case of submental swelling was presented by Wadhera et al. in 2019 [4]. However, the three-year-old patient who presented with a left submental swelling, had no difficulty in swallowing or speech. The patient in our case was a 57-year-old having complaints regarding swallowing and speech and with the swelling present centrally. It implies that the swelling in our patient was a long-standing one, different from the one presented by Wadhera et al. A similar case was noted by Bommaji et al. in 2019 where a 27-year-old male patient reported swelling in the submental region [11]. The patient had a history of impact five years earlier after which the swelling was noted. In our case, the elderly male had no history of trauma in the past and the swelling was present since his childhood.

The submental dermoid cyst is an extremely rare cystic lesion of the oral cavity in an elderly male. It can imitate an enormous plunging ranula in its clinical appearance. Appropriate radiographic and histopathologic evaluation is vital in making the ultimate diagnosis in such situations, as this would help specialists in the precise management of such patients. Initially, the present case was misdiagnosed as a plunging ranula. However, with proper investigations and scans, the diagnosis was confirmed to be a dermoid cyst. 

## Conclusions

The close resemblance of a plunging dermoid cyst to a plunging ranula poses a diagnostic challenge for clinicians. The misdiagnosis of the lesion can lead to inefficient outcomes of the therapy and recurrence of the lesion. Therefore, it is important to carry out all the investigations sequentially to differentiate between the two entities.
